# Biosynthesis of value-added bioproducts from hemicellulose of biomass through microbial metabolic engineering

**DOI:** 10.1016/j.mec.2022.e00211

**Published:** 2022-10-18

**Authors:** Biao Geng, Xiaojing Jia, Xiaowei Peng, Yejun Han

**Affiliations:** a National Key Laboratory of Biochemical Engineering, Institute of Process Engineering, Chinese Academy of Sciences, Beijing, 100190, China; b School of Chemical Engineering, University of Chinese Academy of Sciences, Beijing, 100049, China

**Keywords:** Hemicellulose, Microorganism, Carbon catabolite repression, Metabolic engineering, Bioprocess

## Abstract

Hemicellulose is the second most abundant carbohydrate in lignocellulosic biomass and has extensive applications. In conventional biomass refinery, hemicellulose is easily converted to unwanted by-products in pretreatment and therefore can't be fully utilized. The present study aims to summarize the most recent development of lignocellulosic polysaccharide degradation and fully convert it to value-added bioproducts through microbial and enzymatic catalysis. Firstly, bioprocess and microbial metabolic engineering for enhanced utilization of lignocellulosic carbohydrates were discussed. The bioprocess for degradation and conversion of natural lignocellulose to monosaccharides and organic acids using anaerobic thermophilic bacteria and thermostable glycoside hydrolases were summarized. Xylose transmembrane transporting systems in natural microorganisms and the latest strategies for promoting the transporting capacity by metabolic engineering were summarized. The carbon catabolite repression effect restricting xylose utilization in microorganisms, and metabolic engineering strategies developed for co-utilization of glucose and xylose were discussed. Secondly, the metabolic pathways of xylose catabolism in microorganisms were comparatively analyzed. Microbial metabolic engineering for converting xylose to value-added bioproducts based on redox pathways, non-redox pathways, pentose phosphate pathway, and improving inhibitors resistance were summarized. Thirdly, strategies for degrading lignocellulosic polysaccharides and fully converting hemicellulose to value-added bioproducts through microbial metabolic engineering were proposed.

## Highlights

• Hemicellulose is the main carbohydrate of biomass and has valuable applications.
• Hemicellulose is underutilized in conventional biomass refinery and pretreatment.
• Microbial and enzymatic catalysis were applied for hemicellulose utilization.
• Xylose is converted to value-added bioproducts by metabolic engineering.

## Abbreviations

CCR: Carbon Catabolite Repression
CAP: Catabolite gene-activator protein
AC: Adenylate cyclase
HPrK/P: HPr kinase/phosphorylase
CcpA: Catabolite control protein A
PEP: Phosphoenolpyruvate
PTS: Carbohydrate phosphotransferase system
ABC: ATP-binding cassette
PK: Phosphoketolase pathway
TCA: Tricarboxylic acid cycle
xylO: xyl operator
CoA: Coenzyme A
EMP: Embden-Meyerhof-Parnas

## 1 Introduction

The extensive use of fossil fuels has brought challenges such as environmental pollution and carbon dioxide emission to the world today (Jeffries, 2006). Lignocellulose is the most abundant renewable resource on earth, and converting it to value-added products offers a potential solution to the challenges (Lin and Tanaka, 2006; Brat and Boles, 2013; Jia and Han, 2019; Winkelhausen and Kuzmanova, 1998; Chun et al., 2006). Lignocellulose is mainly composed of cellulose, hemicellulose, and lignin, the polysaccharides can be degraded into hexose (glucose) and pentose (xylose, arabinose) through chemical or biological processes (Cardona and Sanchez, 2007; Zhao et al., 2016; Galbe and Zacchi, 2012). Xylose is the main component of hemicellulose and the second most abundant sugar in nature after glucose (Zhao et al., 2020; Kawaguchi et al., 2006). Xylose is widely used in food (Sasanam et al., 2021; Corim Marim and Gabardo, 2021), medicine (Zhou and Murphy, 2010; Cheudjeu, 2020; Chen et al., 2020), and chemical industry (Zhang et al., 2022a; Wang et al., 2022) as food additive (Sasanam et al., 2021; Corim Marim and Gabardo, 2021), drug excipients (Cheudjeu, 2020), and feedstock (Zhou and Murphy, 2010; Chen et al., 2020; Zhang et al., 2022a; Wang et al., 2022). In addition, xylose can also be used as carbon and energy for microbial metabolism and growth. However, hemicellulose of biomass has not been fully converted to value-added products due to insufficient pretreatment of lignocellulose, carbon catabolic repression (CCR) effect and incomplete understanding of pentose metabolic process. Due to the complexity and recalcitrance structure of natural lignocellulose, it can’t be degraded directly by most microorganisms. Therefore, lignocellulose is usually pretreated by chemical or physicochemical methods in biomass refinery process (Qasim et al., 2021; Yan et al., 2022). While in the pretreatment process, hemicellulose will inevitably be converted to unwanted chemicals (such as furfural, phenol, formic acid etc.) (Guo et al., 2022; Zhang et al., 2022b). It not only causes a waste of resources but also affects the subsequent microbial fermentation and bioproducts synthesis (Hou et al., 2019). Therefore, it’s necessary to develop green and efficient processes to fully convert hemicellulose to monosaccharides and desired bioproducts.

A wide variety of microorganisms in nature can grow with hemicellulose through different metabolic pathways (Jia et al., 2018). Xylose typically coexists with glucose in lignocellulosic hydrolysate, so the CCR effect affects xylose utilization in microbial fermentation. Transmembrane transport is the first and critical step in xylose assimilation in microorganisms, while some of them lack efficient transmembrane transport systems, thus hindering their utilization. Metabolic engineering has been conducted to convert xylose to value-added bioproducts through microbial and enzymatic catalysis (Kim et al., 2010; Singh et al., 2008a; Jia et al., 2017; Ostergaard et al., 2000).

The present review aims to discuss the latest research progress in bioprocess and metabolic engineering to convert hemicellulose to value-added bioproducts. Firstly, microbial metabolic engineering and bioprocess for utilization of lignocellulosic carbohydrates were discussed. Secondly, the metabolism, regulatory, transmembrane transport, CCR mechanism for xylose utilization in microorganisms, and microbial metabolic engineering for converting xylose to value-added bioproducts were summarized. Thirdly, bioprocess for biodegrading biomass polysaccharides and converting hemicellulose to value-added bioproducts through metabolic engineering were proposed.

## 2 Microbial metabolic engineering for enhanced utilization of lignocellulosic carbohydrates

### 2.1 Degradation of lignocellulosic carbohydrates with thermophilic bacteria and thermostable enzymes

Compared with chemical or physicochemical methods, pretreatment of lignocellulose with microorganisms such as fungi is a mild and clean way (Meenakshisundaram et al., 2021). While the efficiency of pretreatment with fungi is comparatively low, and the cost of polysaccharides saccharification with fungi enzyme cocktails is high, which limits the application of fungi in lignocellulose refining (Saini and Sharma, 2021). It has been reported that the thermophilic anaerobic bacteria *Caldicellulosiruptor* can grow at 85゜C with un-pretreated lignocellulose as substrate. Both cellulose and hemicellulose of natural biomass can be assimilated, and interestingly lignin can also be partially solubilized by *Caldicellulosiruptor* (Peng et al., 2018; Brunecky et al., 2013).

The genes encoding glycoside hydrolases (GHs) for cellulose and hemicellulose degrading have been identified in the genome of *Caldicellulosiruptor*, and an increasing number of GHs have been expressed and characterized from different *Caldicellulosiruptor* strains (Table 1). Some exo- and endo-GHs from *Caldicellulosiruptor* have evolved multiple domain modules, such as S-layer homology (SLH) domain, catalytic domain, carbohydrate-binding modules (CBMs), and linker peptides (Jia et al., 2014; Mi et al., 2014). Compared with the enzymes from mesophilic fungi and thermophilic bacteria, the thermostable GHs from *Caldicellulosiruptor* show the merits of higher activity and stability and are active on un-pretreated lignocellulose (Jia et al., 2016). In addition, by using the heterologously expressed thermostable GHs from *Caldicellulosiruptor*, enzyme cocktails have been constructed and successfully applied for cellulose and hemicellulose degradation (Jia and Han, 2019; Peng et al., 2018; Su et al., 2013; Qiao et al., 2014).

**Table 1:** Glycoside hydrolases identified in different *Caldicellulosiruptor* strains.

Bacteria	Glycoside hydrolase type	Substrates	References
C*aldicellulosiruptor* bescii	*β*-Xylosidase(Xyl3A)	xylan	Su et al. (2013)
C*aldicellulosiruptor* bescii	*β*-mannosidase (CbMan2A)	mannooligosaccharides	Liang et al. (2015)
C*aldicellulosiruptor* bescii	Glucosidase (CbBgl1A)	A range of cellooligosaccharides and aryl-*β*-glycosides	Bai et al. (2013)
C*aldicellulosiruptor* Saccharolyticus DSM 8903	Recombinant *β*-glucosidase	*ρ*-nitrophenyl (pNP)-*β*-D-glucopyranoside and *ρ*NP-*β*-D-fucopyranoside, etc.	Hong et al. (2009)
C*aldicellulosiruptor owensensis*	Recombinant *β*-glycosidase	Platycoside E	Shin et al. (2019)
C*aldicellulosiruptor owensensis*	GH10 endo-*β*-1,4-xylanase (Coxyn A)	Beechwood xylan	Mi et al. (2014)
	GH39 *β*-1,4- xylosidase (Coxyl A)	*ρ*NP-*β*-D-xylopyranoside	
C*aldicellulosiruptor* bescii DSM 6725	GH family 10 xylanase (CbXyn10B)	Beech wood xylan, oat spelt xylan, and birch wood xylan, etc.	An et al. (2015)
C*aldicellulosiruptor* bescii	Bifunctional glycoside Hydrolase (CelA)	–	Young et al. (2014b)
C*aldicellulosiruptor* Saccharolyticus	*β*-Glucosidase A (BglA)	–	Hardiman et al. (2010)
C*aldicellulosiruptor* kronotskyensis	GH11 xylanase (Xyn11A)	Beechwood xylan	Qiao et al. (2014)
C*aldicellulosiruptor* kronotskyensis	Pectate lyase Pel-863	Polygalacturonic acid, methylated pectin and pectic biomass, etc.	Su et al. (2015)
C*aldicellulosiruptor* lactoaceticus 6A	GH family 10 xylanase (Xyn10A)	beechwood xylan	Jia et al. (2014)
	GH67 α-glucuronidase (Agu67A)	xylo-oligosaccharides	
C*aldicellulosiruptor* lactoaceticus 6A	bifunctional acetyl ester−xyloside hydrolase (CLH10)	*ρ*-Nitrophenyl-*β*-Dxylopyranoside (*ρ*NPX) and *ρ*-nitrophenyl acetate (*ρ*NPA)	Cao et al. (2019)
*Caldicellulosiruptor* sp. F32	xylanase JX030400	0.5% (w/v) xylan	Ying et al. (2013)
	Xylanase JX030401	0.5% (w/v) xylan	

**Fig. 1:**
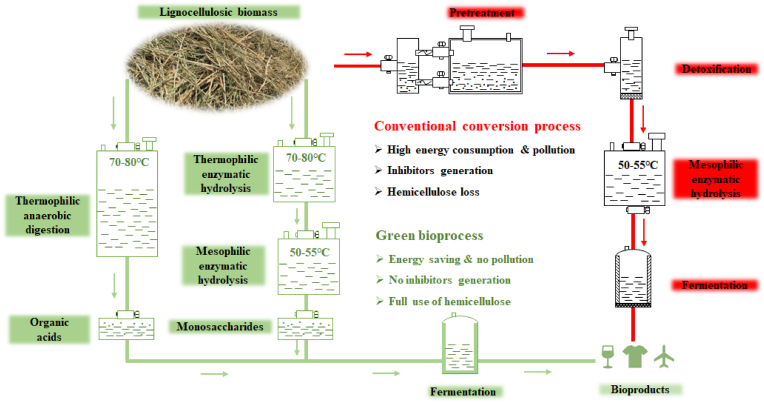
Bioprocess for converting polysaccharides of unpretreated lignocellulosic biomass to monosaccharides and organic acids. In the bioprocess, the un-pretreated lignocellulose is sequentially hydrolyzed with thermostable enzymes and then mesophilic enzymes, the produced monosaccharides were used for fermentation. Alternately, the unpretreated lignocellulose can be digested with thermophilic anaerobic bacteria, and the generated organic acids were used for fermentation.

When *Caldicellulosiruptor* was cultured with un-pretreated lignocellulose as substrate, both extracellular and intracellular GHs were identified and characterized. A bioprocess for converting natural lignocellulose to monosaccharides by sequential hydrolysis with extracellular GHs of *Caldicellulosiruptor* and commercial cellulase from fungi was developed. With the bioprocess, the unpretreated lignocellulosic biomass was successfully degraded to monosaccharides by sequential enzymatic hydrolysis. The lignocellulose degradation performance of the sequential bioprocess is comparable with that of the traditional process combining chemical or physicochemical pretreatment and enzymatic hydrolysis. With the sequential bioprocess, hemicellulose of biomass can be fully utilized without being converted to un-wanted chemicals (Peng et al., 2015, 2016; Li et al., 2019). In addition to the application of thermostable GHs, *Caldicellulosiruptor* has also been combined with *Cupriavidus necator* for converting rice straw to polyhydroxybutyrate. Un-pretreated rice straw was degraded and converted to organic acids, hydrogen, and lignin-derived aromatics by *Caldicellulosiruptor* under thermophilic and anaerobic fermentation (Peng et al., 2018; Soto et al., 2019). The chemicals produced by the anaerobic digestion with *Caldicellulosiruptor* were assimilated and converted to polyhydroxybutyrate by *C. necator* (Peng et al., 2018) (Fig. 1). It has been reported that cellulose, hemicellulose, and lignin of unpretreated switchgrass were simultaneously solubilized by Caldicellulosiruptor bescii, and 85% of insoluble biomass was degraded at 78 °C (Kataeva et al., 2013)*.* The lignocellulose solubilization was further promoted by maintaining the bioreactor culture in a metabolically active state of *Caldicellulosiruptor bescii* (Straub et al., 2019). In addition to *Caldicellulosiruptor*, natural biomass can also be anaerobically degraded and converted to organic acids by the biogas fermentation bacteria and *Clostridium* (Yang et al., 2022; Du et al., 2020; Ji et al., 2022).

### 2.2 Xylose transmembrane transport in microorganisms

#### 2.2.1 Xylose transmembrane transportation in natural microorganisms

Xylose is assimilated by microorganisms through specific or non-specific transmembrane transporting systems (Paulsen et al., 1998). There are two specific xylose transporting systems in bacteria:the ABC transportation system (ATP-binding cassette, ABC) and proton/cation-linked transport systems (Wang et al., 2018; Wambo et al., 2017). The ABC transportation system mainly exists in *E. coli*, *Haloferax volcanii*, and some rumen bacteria (Luo et al., 2014; Johnsen et al., 2019). In the ABC transporter system, xylose is transported through specific xylose binding proteins driven by the energy produced from ATP hydrolysis. The proton/cation-linked transport systems are mainly present in *Lactobacillus brevis*, *Salmonella*, and some *Bacillus*. In the system, xylose is transported by transmembrane transport proteins and driven by Na^+^ concentration gradient or proton power potential (Jojima et al., 2010).

The assimilation of xylose in fungi is mainly mediated by a high-affinity glucose transport system, so the presence of glucose will strongly inhibit the utilization of xylose (Nijland et al., 2019; Meinander and Hahn-Hagerdal, 1997). The uptake of xylose and glucose in yeast mainly depends on the proton-driven cotransport systems (Gardonyi et al., 2003). There are two modes identified in *Pichia stipitis* CBS7126 for xylose transport, which are high affinity and low-affinity systems. The low-affinity transport system is shared by glucose and xylose, while the high-affinity transport system of xylose is non-competitively inhibited by glucose (Hahn-Hägerdal et al., 2001; Nobre et al., 1999). Unlike *Pichia* yeast, *Candida shehatae* CBS 2779 has a facilitated diffusion system (FDS) for both xylose and glucose besides the proton-driven cotransport systems. *S. cerevisiae* can use xylose under certain conditions, which is mainly transported through FDS (Nobre et al., 1999), while the affinity is much lower than that of glucose (Reider Apel et al., 2016; Sharma et al., 2018). The xylose transport system of *B. subtilis* belongs to proton-driven transport systems, and the transmembrane transport protein is AraE (Kim et al., 2017; Park et al., 2012). The AraE of *B. subtilis* has an affinity for xylose, arabinose, and galactose, so the transportation system is shared by three monosaccharides (Park et al., 2012; Krispin and Allmansberger, 1998; Mota et al., 1999).

#### 2.2.2 Improving xylose transmembrane transport in microorganisms by metabolic engineering

Transmembrane transportation is the key step for xylose utilization, therefore improving the efficiency of xylose transmembrane transport is an effective way to increase its utilization and expand carbon sources of microorganisms (Dien et al., 2002). For example, the absorption of xylose in yeast is mainly mediated by high-affinity glucose transport factors, so the presence of glucose will strongly inhibit xylose absorption. Although xylose can be transported across the membrane, the affinity is 200 times lower than that of glucose (Kawaguchi et al., 2006; Meinander and Hahn-Hagerdal, 1997). Similarly, xylose is transported through glucose-facilitated diffusion protein in *Z. mobilis*, which also limits the transmembrane transport of xylose (Sarkar et al., 2021). As the most common microorganism for lignocellulosic hydrolysates fermentation, much effort has been made to increase xylose transmembrane transport and co-utilization in *S. cerevisiae*. The assimilation of xylose in *S. cerevisiae* is mainly mediated by the high-affinity glucose transport factors HXT4, HXT5, HXTZ, and GAL2, the presence of glucose strongly inhibits xylose transport (Hou et al., 2017; Jeffries and Jin, 2004). The high glucose transporter in *S. cerevisiae* was therefore modified to increase xylose affinity and transmembrane transport. By replacing the N-terminal tail of the high-affinity glucose transporter HXT2 with the corresponding region of HXT11, the resulting HXT11/2 transporter can effectively assimilate xylose even at high glucose concentrations (Shin et al., 2017). When the phenylalanine at position 79 of HXT7 was mutated to serine, the affinity of glucose was decreased and the affinity of xylose was increased (Reider Apel et al., 2016). Through reshuffling the genes of the highly homologous hexose transporter family by using the hexose transporter-deficient strain *S. cerevisiae* DS68625 as a host, the xylose affinity of mutant Hxt2C505P was increased and provided a reference to promote xylose transport under low concentration (Nijland et al., 2018). Young et al. found that the specific sequence of GG/F-XXX-G motif in CiGXS1 transporter (V38F, L39I, F40M) can attenuate glucose transport without impairing xylose transport (Young et al., 2014a). Through directed evolution of CiGXS1 transporter in *S. cerevisiae*, several mutations including N326H, C-terminal truncation, I171F, and M40V were found to reduce glucose inhibition. The transport of xylose in the presence of a high concentration of glucose (up to 70 g/L) was significantly improved in the mutant *S. cerevisia* (Li et al., 2016). To promote xylose assimilation, xylose transporters from bacteria and fungi have also been introduced into yeast to improve xylose transport. The xylose cotransporters Gxf1 and Sut1 from *Candida intermedia* and *Pichia stipitis* were integrated into *S. cerevisiae*, and it was found that xylose transport and affinity were increased (Runquist et al., 2010). Through integrating *B. subtilis* AraE gene into *S. cerevisiae* D452-2, the xylose transport and xylitol production were increased significantly (Kim et al., 2017). The gene of XltA from *Aspergillus nigerda* was introduced into *S. cerevisiae* and the xylose utilization was increased (Sloothaak et al., 2016).

In addition to yeast, efforts have also been made to elucidate the xylose transporting mechanism and improve xylose transport in bacteria. The xylan metabolism and transmembrane transport in the thermophilic bacterium *Caldanaerobius polysaccharolyticus* were studied. The solute-binding protein XBP1 of ABC transporter in *C. polysaccharolyticus* was characterized and the co-crystal structure of XBP1 was solved (Han et al., 2012). Erbeznik et al. analyzed the xylFGH operon and xylose ABC transporter in *Thermoanaerobacter ethanolicus* (Erbeznik et al., 2004). Though introducing a xylose transporter (ABC type transporter system) into *Z. mobilis*, xylose utilization of which was increased by 48.9%, and the fermentation time was greatly shortened (Sarkar et al., 2021). The xylose/arabinose transport in *Sulfolobus acidocaldarius* via a 2 (CUT2)-Type ABC transporter, which is the first CUT2 family ABC transporter analyzed in the domain of Archaea (Wagner et al., 2018).

### 2.3 Co-utilization of glucose and xylose in microorganisms by overcoming the CCR effect

#### 2.3.1 CCR effect and xylose utilization in microorganisms

The main physiological phenomenon of CCR is that when glucose is present, microorganisms only synthesize the enzymes for glucose metabolism instead of other sugars. It has been found that some intermediates produced from glucose can inhibit the transcription of genes for other sugars' metabolism (Lu et al., 2016; Vinuselvi et al., 2012). CCR is a global regulatory mechanism of bacteria, and about 5–10% of genes in bacteria are regulated by CCR (Kurgan et al., 2019).

In the CCR regulation of *E. coli*, cyclic AMP receptor protein(CRP) also called catabolite gene-activator protein(CAP), the signal metabolite cAMP, adenylate cyclase (AC), and EIIAGlc of glucose transporter are involved in the process (Vinuselvi et al., 2012; Singh et al., 2008b) (Fig. 3). When the medium does not contain glucose, the phosphorylated EIIAGlc can activate adenylate cyclase and promote the synthesis and accumulation of intracellular cAMP. High concentrations of cAMP will bind to CRP to form a CRP complex, which can combine with the promoter for RNA synthetase, thereby promoting the transcription of genes for other carbohydrates catabolism (Wang et al., 2018; Lu et al., 2016; Warner and Lolkema, 2003; Görke and Stülke, 2008). The CCR mechanism in bacilli and other Gram-positive bacteria with low GC content is different from *E. coli*. The major regulatory protein of CCR is catabolite control protein A (CcpA), and it has been reported that about 10% of gene transcription in *B. subtilis* is regulated by CcpA (Zhang et al., 2016). CcpA binds to catabolite responsive elements (cre) boxes to mediate CCR, and CcpA proteins from different microorganisms are highly conserved (Wang et al., 2018; Zhang et al., 2016). The cre boxes are a 14 bp palindrome sequence with a common sequence of TGWNANCGNTNWCA (W: A/T, N: A/G/C/T) (Han et al., 2020). In CCR regulation, CcpA binds the cre site through the complex of CcpA-HPr-Ser-P or CcpA-Crh-Ser-P, which are formed by combination with phosphoproteins of histidine-containing protein(HPr) and catabolite repression HPr-like protein(Crh). Crh and HPr are structurally homologous, CcpA binds HPr-Ser-P stronger than Crh-Ser-P, and HPr-Ser-P is more commonly used in CCR regulation. The structure of both CcpA-(HPr-Ser-P)-cre complex and CcpA-(Crh-Ser-P)-DNA complex has been determined (Schumacher et al., 2006). Besides bacteria, CCR also exists in yeasts, fungi, and metazoa to prevent the utilization of alternative sugars in presence of glucose. In *S. cerevisiae*, three signaling pathways of CCR have been observed, including inhibition of AMPKSnf1, activation of PKA, and regulation of expression and stability of transporter by casein kinases (Soontorngun, 2017; Simpson-Lavy and Kupiec, 2019).

#### 2.3.2 Eliminating CCR in microorganisms by metabolic engineering

The existence of CCR effect in microorganisms severely restricts the absorption and metabolism of xylose, so efforts have been made to reduce or eliminate CCR effect. Three strategies have been applied to address the CCR effect and promote xylose utilization, namely disrupting signaling pathways of CCR effect, engineering xylose transport system, and constructing a microbial co-culture system.

The CCR signaling pathway of *E.coli* was weakened through disrupting *pts*G gene of phosphotransferase system (PTS). After being cultured for 48 h, the wild strain consumed only 12.5% xylose after consuming 37.5% glucose, while the mutant strain can consume 37.5% glucose and 37.5% xylose to produce PHA_SCL_ (Li et al., 2007). In addition, a mutant *E.coli* was constructed for xylitol production by deleting *ara*C and expressing genes of xylose metabolism, and xylose and glucose were co-utilized with CCR elimination (Kim et al., 2015a). To co-utilize glucose and xylose, the xylose transporters without inhibition by glucose were engineered in *S. cerevisiae* (Farwick et al., 2014). Through deleting the gene of D-ribulose-5-phosphate 3-epimerase (RPE1) to reduce the conversion of glucose to xylose-5-phosphate and constructing PP pathway, simultaneous utilization of xylose and glucose was realized in *S. cerevisiae* (Shen et al., 2015). To co-utilize cellobiose, xylose, and acetic acid for ethanol production with *S. cerevisiae*, the integrated pathways for glucose and xylose metabolism and acetic acid reduction were constructed, and the ethanol yield was much higher than the control strain (Wei et al., 2015).

To co-utilize glucose and xylose in *C. tyrobutyricum* ATCC 25755 (CT), a recombinant strain Ct-pTBA was constructed through the over-expression of genes *xyl*T, *xyl*A, and *xly*B from *C. acetobutylicum* ATCC 824. Compared with the parent strain(CT), the engineered strain Ct-pTBA showed a higher utilization rate of xylose (1.28 g/L·h vs 0.16 g/L·h) in the co-existence of glucose, and also produced more butyric acid (0.53 g/L·h vs 0.26 g/L·h) (Fu et al., 2017). The mechanism of CCR in *Streptomyces avermitilis* was studied, and the ROK-family regulator Rok7B7 regulates xylose and glucose uptake was identified. Through deleting gene rok7B7 in *S. avermitilis*, a mutant strain with an in-frame deletion of rok7B7(Δrok7B7) was constructed. At 72 h, the parent strain consumed 33% glucose and 4% xylose, the mutant strain(Δrok7B7) consumed 25% glucose and 38% xylose, and the production of avermectin and oligomycin A were increased (Lu et al., 2020).

To alleviate CCR and enhance the utilization of glucose and xylose, a co-culture system of *Clostridium beijerinckii* and *S. cerevisiae* was developed. In the co-culture system, xylose and glucose were co-utilized, and the xylose consumption was increased by 32.99% compared with the single culture system of *C. beijerinckii* F-6 (Wu et al., 2020). In the fermentation of thermophilic *Enterococcus faecium* QU 50 for lactic acid production, CCR was observed when glucose and xylose mixtures were applied and were eliminated when cellobiose was fed (Abdel-Rahman et al., 2020). To overcome CCR and biosynthesis of glycolic acid with xylose as substrate, a metabolically engineered *E.coli* was constructed. Cellobiose phosphorylase was overexpressed in *E.coli* for cellobiose utilization, and the glyoxylate shunt pathway was activated. By using the engineered *E.coli*, glycolic acid production reached the maximum theoretical yield with xylose and cellobiose as substrates (Cabulong et al., 2021).

## 3 Synthesis of value-added bioproducts from xylose through metabolic engineering

### 3.1 Metabolic pathways of xylose utilization in microorganisms

**Fig. 2:**
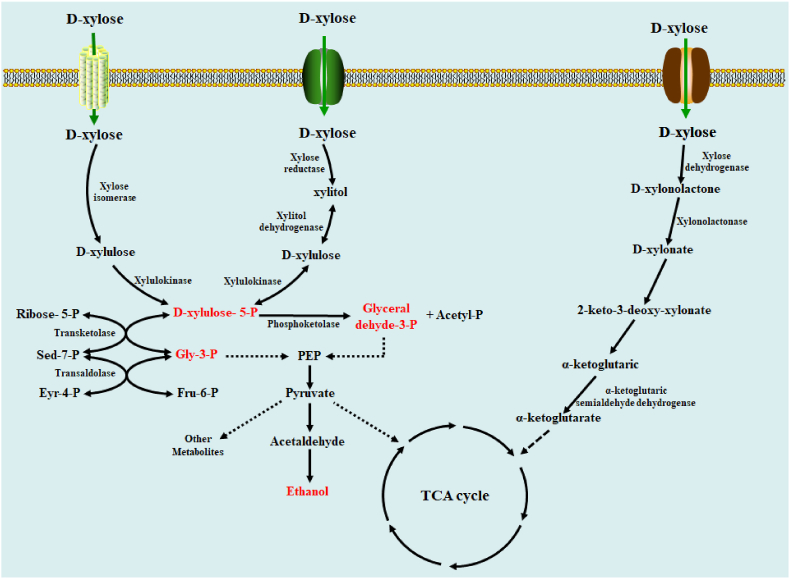
Metabolic pathways for xylose utilization in microorganisms. The pathways for xylose catabolism in microorganisms include the xylose reductase-xylitol dehydrogenase (XR-XDH) pathway, isomerase pathway, pentose phosphoketolase (PK) pathway, weimberg pathway, and dahms pathway. Sed-7-P: Sedoheptulose-7-P; Gly-3-P: Glyceraldehyde-3-P; Eyr-4-P: Erythrose-4-P; Fru-6-P: Fructose-6-P; PEP: Phosphoenolpyruvate.

As the main component of hemicellulose, microorganisms have evolved specific pathways for xylose catabolism, including xylose reductase-xylitol dehydrogenase (XR-XDH) pathway, isomerase pathway, pentose phosphoketolase (PK) pathway, weimberg pathway, and dahms pathway (Fig. 2) (Cheudjeu, 2020; Robak and Balcerek, 2020). Most fungi and yeasts employ the XR-XDH pathway for xylose catabolism, xylose reductase (XR) and xylitol dehydrogenase (XDH) are the two characteristic enzymes of the catabolism (Zhao et al., 2020; Nijland et al., 2019; Han et al., 2012; Kerstin and Bärbel, 1988). Isomerase pathway for xylose catabolism has been identified in bacteria such as *E. coli*, *Bacillus*, and *Lactobacillus*, some fungi (Zhou et al., 2012), and plants (Maehara et al., 2013). The isomerase pathway is similar to XR-XDH pathway except for the reaction of converting xylose to xylulose. In the pathway, xylose is directly catalyzed to xylulose by xylose isomerase without the participation of coenzymes (Zhao et al., 2020; Hou et al., 2017; Nijland et al., 2017). Pentose phosphoketolase(PPK) pathway (Fig. 2) is a major route of glucose and xylose catabolism and is characterized by phosphoketolase, which is mainly identified in lactic acid-producing bacteria and *Clostridium* (Zhao et al., 2020; Jeffries, 1983). Through quantification analysis, phosphoketolase pathway was found to attribute up to 40% of xylose catabolism in *Clostridium acetobutylicum* (Liu et al., 2012). Weimberg pathway was identified in *Pseudomonas fragi*, *Caulobacter crescentus*, *Halophilic archaea*, and so on (Zhao et al., 2020; Johnsen et al., 2019). Dahms pathway is similar to Weimberg pathway except that 2-keto-3-deoxy-xylonate is catalyzed to pyruvate and glycolaldehyde by keto-3-deoxy-xylonate (KDX)-aldolase (Zhao et al., 2020).

**Fig. 3:**
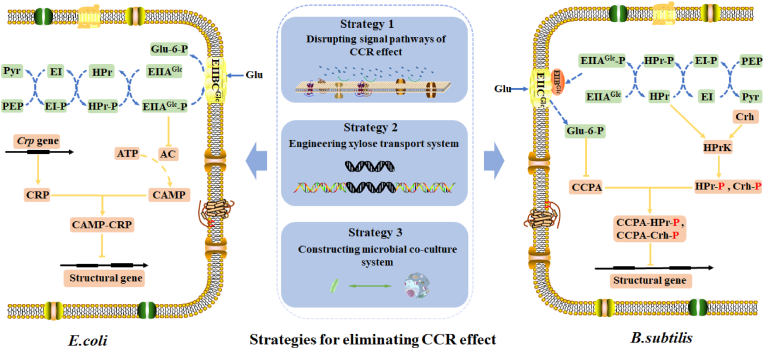
Carbon catabolite repression effect and eliminating CCR in microorganisms by metabolic engineering. The molecular basis of CCR effect in *E.coli* and *B.subtilis* has been clarified. Metabolic engineering strategies for eliminating the CCR effect have been developed, including disrupting signal pathways of CCR effect, engineering a xylose transport system, and constructing a microbial co-culture system. Glu: glucose; PEP: phosphoenolpyruvate; Pyr: Pyruvate; AC: adenylate cyclase; CRP: cyclic AMP receptor protein; cAMP: cyclic AMP; HPr: Histidine protein; EI: enzyme I; Glu- 6-p: glucose-6-phosphate; EIIAGlc, EIIBGlc, EIICGlc, EIIBCGlc: EIIA, EIIB, EIIC and EIIBC domains of the glucose transporter. Crh: catabolite repression HPr-like protein; HPrK: HPr kinase; CCPA: Catabolite control protein A; HPr-P, Crh-p: HPr-Ser46-P and Crh-Ser46-P.

### 3.2 Metabolic engineering of microorganisms for value-added bioproducts synthesis from xylose

#### 3.2.1 Microbial metabolic engineering based on redox pathways

Microbial metabolic engineering through XR-XDH pathways: The XR-XDH pathway has been introduced into *S. cerevisiae* to convert lignocellulosic hydrolysates to ethanol (Table 2). By expressing XK, XR, and XDH of *S. stipitis* in *S. cerevisiae*, xylose consumption and ethanol production of the engineered *S. cerevisiae* HX57D were significantly improved (Xie et al., 2020). The production of xylitol was catalyzed by XR, while the catalysis was repressed in *Candida tropicalis* fermentation with the presence of glucose. The codon of XR from *Neurospora crassa* was optimized and expressed in *C. tropicalis* under a constitutive promoter, and xylose utilization was not repressed by glucose in the engineered strain. The xylitol yield of the recombinant *C. tropicalis* reached 1.44 g/(L·h) compared with 0.83 g/(L·h) of the original strain (Jeon et al., 2012). Both glucose and xylose can be converted to xylitol and ethanol by *Kluyveromyces marxianus* IIPE453. To increase xylitol production further, the endogenous XR gene was overexpressed in *K. marxianus*, and the yield of xylitol was increased by 1.62 times with no effect on ethanol production (Dasgupta et al., 2019). When XR and xylitol dehydrogenase genes from *Spathaspora passalidarum* were overexpressed in *Aureobasidium pullulans*, xylose consumption increased by 17.76% compared with the parent strain. In addition, the fermentation of engineered *A. pullulans* with xylose as the carbon source was not affected by the presence of glucose (Guo et al., 2018).

**Table 2:** Summary of xylose metabolism engineering.

Metabolic engineering		Modified strains	Gene/protein modification	Substrates	Reaction rate	References
Transmembrane transport of xylose		*S. cerevisiae* DS68616	N-terminal tail of Hxt2	D-glucose and D- xylose	–	Shin et al. (2017)
		*S. cerevisiae* BY4742	Position 79 of HXT7	D-xylose	186.4 ± 20.1 nmol/(min•mg)	Reider Apel et al. (2016)
		*S. cerevisiae* DS68625	Position 505 in Hxt2	D-glucose and D-xylose	–	Nijland et al. (2018)
		*S. cerevisiae* EBY.VW4000	CiGXS1 FIM transporter	D-xylose	–	Li et al. (2016)
		*S. cerevisiae strains* TMB 3043	Gxf1, Sut1 and At5g59250	D-glucose and D-xylose	–	Runquist et al. (2010)
		*S. cerevisiae* D452-2	AraE	D-xylose	–	Kim et al. (2017)
		*S. cerevisiae* EBY.VW4000	XltA	D-xylose	–	Sloothaak et al. (2016)
		*C. polysaccharolyticus*	Solute-binding protein XBP1	xylan	–	Han et al. (2012)
		*Thermoanaerobacter ethanolicus* ATCC 33223	xylFGH operon and xylose ABC transporter	D-xylose	–	Erbeznik et al. (2004)
		*Zymomonas mobilis* AD50	ABC type transporter system	D-glucose and D-xylose	–	Sarkar et al. (2021)
		*Sulfolobus acidocaldarius*	Cut2-type ABC transporter	D-Xylose and L-arabinose	–	Wagner et al. (2018)
Eliminate CCR effects		*E. coli* MG1655	PtsG	D-xylose	–	Li et al. (2007)
		*E. coli* MG1655	araC and Pentose metabolism pathway	D-xylose	–	Kim et al. (2015a)
		*S. cerevisiae* L2612	RPE1 and PP pathway	D-glucose and D-xylose	–	Shen et al. (2015)
		*S. cerevisiae* D452-2	Fermentation pathways for hexose and pentose sugars and acetic acid reduction	Cellobiose, xylose and acetic acid	–	Wei et al. (2015)
		*C. tyrobutyricum* ATCC 25755	XylT, XylA, and XlyB	D-xylose	1.28 g/L·h	Fu et al. (2017)
		*Streptomyces avermitilis*	Rok7B7	D-xylose	–	Lu et al. (2020)
		*Clostridium beijerinckii* F-6 and *S. cerevisiae*	–	Glucose, xylose and arabiose	37.77 g/L	Wu et al. (2020)
		*Enterococcus faecium* QU 50	–	Glucose, cellobiose and xylose	5.0 ± 0.0, 39.9 ± 0.705, 18.7 ± 0.45 g/L	Abdel-Rahman et al. (2020)
		*E. coli* CLGA8	*cep94A*,XDH	D-Xylose and cellobiose	–	Cabulong et al. (2021)
Xylose metabolism pathway	Redox pathways	*Candida tropicalis*	XR (*Neurospora crassa*)	D-xylose	–	Jeon et al. (2012)
		*Kluyveromyces marxianus* IIPE453	XR (native strain)	D-xylose	0.449 g/ L·h	Dasgupta et al. (2019)
		*Aureobasidium pullulans* CBS 110374	XR,XDH (*Spathaspora passalidarum*)	D-xylose	48.73 ± 0.49 g/L	Guo et al. (2018)
		*S. cerevisiae* CEN.PK2	XR、XDH and XK (P. stipitis), GDP1 and ZWF1	D-xylose	0.32 g/ L·h	Verho et al. (2003)
		*S. cerevisiae* TMB3001c	ACDH (*E. histolytica*), Phosphoketolase (*B. lactis*) and *pta* (B. subtilis)	D-xylose	0.17 g/ g·h	Sonderegger et al. (2004)
		*S. cerevisiae*	xylD, xylX and xylB(*Caulobacter crescentus*), KsaD (Corynebacterium glutamicum) and FRA2	D-xylose	–	Borgstrom et al. (2019)
		–	Xdh, XylC, YagF, YjhH, FucO, ALS2 and ALDC	D-xylose	–	Jia et al. (2018)
		*E. coli*	XylBCccs	D-xylose	–	Choi et al. (2017)
	Non-redox pathways	*S. cerevisiae* 2805*Δgal80*	XI (*Piromyces* sp.)	D-glucose and D-xylose	60 ± 0.8 and 65.3 ± 2.6 g/L	Bae et al. (2021)
		*Mucor circinelloides*	XI and XK	D- xylose	–	Chu et al. (2016)
	Pentose phosphate pathway	*S. cerevisiae* YUSM1009a	GRE3, XI, XK, TAL, TKL, RKI, RPE	D-xylose	–	Karhumaa et al. (2005)
		*S. cerevisiae* IR-2	TAL1, TKL1, RKI1 and RPE1(Sc, Km)	D-xylose	1.79 g/ L·h (30 °C) and 1.61 g/ L·h (36 °C)	Kobayashi et al. (2017)
		*S. cerevisiae* CEN.PK2−1C	TAL1, STB5, TKL1, DDGS, OMT, ATP-grasp ligase and _D_-ala-_D_-ala ligase	D-glucose and D-xylose	–	Park et al. (2019)
		*E. coli* W3110	*zwf* ,*gnd*, *pfkA*, *pfkB*, *pgi*	D-glucose and D-xylose	1.23 g/ L·h and 0.6 g/ L·h	Yuan et al. (2021)
		*Synechococcus elongatus* UTEX 2973	Pkt, Pfk, Fbp, 3-HP biosynthetic pathway	Xylose	–	Yao et al. (2022)
	Inhibitor resistance pathways	*E. coli* SSK42	*pgi*	D-glucose and D-xylose	–	Jilani et al. (2020)
		*S. cerevisiae* D452-2	*OAZ1*, *TPO1*, *ATG7*, *SPE1*, *SPE2*, *SPE3*, *ADE17*, *PIR3* and HTA2	lignocellulosic hydrolysates	–	Kim et al. (2015b)
		*S. cerevisiae*	*ari1*, *tps1*, *nth1*	D-glucose	–	Divate et al. (2017)
		*Synechococcus elongatus* PCC7942 and *Pseudomonas putida*	*cscB*	5-Hydroxymethylfurfural	–	Lin et al. (2020)
		*Raoultella ornithinolytica* BF60	*dcaD*, *aldR*, *aldh1*	5-hydroxymethylfurfural	–	Hossain et al. (2017)
		*Pseudomonas putida* EM42	*dmp* monooxygenase, meta-cleavage pathway, *clpB*, *groES*, and *groEL*	lignocellulose CFP	–	Henson et al. (2021)
		*S. cerevisiae* BY4741	Pdr18	–	–	Teixeira et al. (2012)

Redox balance in microorganisms through metabolic engineering: Redox balance is an important aspect of microbial metabolic engineering of xylose utilization based on the redox pathway (Khattab et al., 2013). A study on *S. cerevisiae* found that introducing coenzyme-related metabolic pathways, reducing the bypass consumption of NADPH, or increasing the utilization of NADH could balance coenzymes and improve metabolic efficiency. An engineered *S. cerevisiae* for converting xylose to ethanol was constructed by expressing XR, XDH, and XK genes of *P. tipitis* and knocking out the 6-phosphate glucose dehydrogenase(ZWFL) gene. To promote NADPH regeneration, the NADP^+^ dependent D-glyceraldehyde-3-phosphate dehydrogenase (NADP-GAPDH) (EC1.2.1.13) from *Kluyveromyces lactis* was also expressed. Compared with the original strain, ethanol production of the engineered *S. cerevisiae* from xylose increased by 50% (Verho et al., 2003). By using the recombinant *S. cerevisiae* harboring XR, XDH, and XK of *P. stipitis* as the starting strain, a phosphoketolase pathway for xylose fermentation was established by expressing a phosphotransacetylase gene from *B. subtilis*, acetaldehyde dehydrogenase gene from *E. histolytica*, and phosphoketolase gene from *B. lactis*, and ethanol production increased by 25% compared with the control strain (Sonderegger et al., 2004).

Microbial metabolic engineering based on Weimberg pathway: Weimberg pathway is an oxidative and non-phosphorylative process, in which xylose was metabolized to α-ketoglutarate. Weimberg pathway in *S. cerevisiae* was constructed by expressing xylD, xylX, xylA of *Caulobacter crescentus*, KsaD of *Corynebacterium glutamicum*, and deleting the FRA2 gene. The engineered *S. cerevisiae* with Weimberg pathway could grow with xylose and be used for biosynthesis with lignocellulosic hydrolysates (Borgstrom et al., 2019). An *in vitro* metabolic engineering was constructed to synthesize (*R*)-acetoin and ethylene glycol from xylose based on Weimberg pathway. The seven-step synthesis with in situ coenzyme regeneration was constructed using xylose dehydrogenase, xylonolactonase, xylonate dehydratase, 2-keto-3-deoxy-D-xylonate aldolase, lactaldehyde reductase, α-acetolactate synthase, and α-acetolactate decarboxylase. In the ATP-free and cell-free synthetic system, 99.2% of xylose was consumed and (*R*)-acetoin with stereoisomeric purity of 99.5% was obtained under the optimum condition (Jia et al., 2018).

Microbial metabolic engineering based on Dahms pathway: An engineered *E. coli* was constructed for poly(d-lactate-co-glycolate) and poly(d-lactate-co-glycolate-co-d-2-hydroxybutyrate) production from xylose by introducing Dahms pathway. The metabolic flux towards Dahms pathway was then modulated, and 6.93 g/L polymer was produced from xylose in fed-batch fermentation of engineered *E. coli* (Choi et al., 2017). A parallel metabolic pathway was constructed in *E. coli* for cis,cis-muconic acid production by using the mixture of glucose and xylose. Through the Dahms pathway, xylose is converted to pyruvate and glyoxylate, which then flow into the 10.13039/100004915TCA cycle and support cell growth. Through metabolic engineering of *E. coli*, glucose was used for cis,cis-muconic acid synthesis, and xylose was used for microbial growth by the introduction of Dahms pathway (Fujiwara et al., 2020).

#### 3.2.2 Microbial metabolic engineering based on non-redox pathways

In addition to the redox pathways described above, most prokaryotes and some fungi directly convert xylose to xylulose by XI pathway with no affection from redox balance. The exogenous XI pathway has been introduced to construct recombinant strains for metabolizing xylose and biosynthesis (Katahira et al., 2017; Hou et al., 2016; Silva et al., 2021). A new XI was recently identified in the goat rumen microbiome and was functionally expressed in *S. cerevisiae*. The recombinant *S. cerevisiae* with the expression of XI can metabolize xylose and grow on media with xylose as the sole carbon source (Colombo et al., 2022). The extracellular conversion of xylose to xylulose by XI before transmembrane transport was also studied in *S. cerevisiae*. The XI activity of *Piromyces* sp. Was improved by directed evolution and then introduced into *S. cerevisiae* for secretory expression. In fermentation with xylose or glucose/xylose, the xylose consumption and ethanol production of the engineered *S. cerevisiae* were improved markedly (Bae et al., 2021).

The XI pathway was identified in the genome of *Mucor circinelloides*, the genes of XI and xylulokinase(XK) were then overexpressed. The results showed that the overexpression of XI or XK increased xylose utilization and lipid accumulation in the engineered *Mucor circinelloides* (Chu et al., 2016). To compare XI and XR/XDH pathways for xylose assimilation, the two pathways were introduced into *S. cerevisiae* separately and simultaneously. Higher ethanol production from xylose was obtained in the engineered *S. cerevisiae* with XI pathway than that of XR/XDH pathway. In contrast, when XR/XDH and XI pathways were simultaneously introduced into *S. cerevisiae*, ethanol production from non-detoxified hydrolysates was improved compared to the single pathway of XR/XDH or XI (Cunha et al., 2019).

Microbial metabolic engineering based on phosphoketolase pathway: In the biosynthesis with xylose as a carbon source, the bioproducts were usually generated using acetyl-CoA as an intermediate. A molecular carbon dioxide was generated in the process of pyruvate decarboxylation to acetyl-CoA, therefore pyruvate can not be fully converted to bioproducts. In the phosphoketolase pathway of xylose metabolism, acetyl-CoA is generated by phosphoketolase catalysis without carbon loss through decarboxylation. Therefore, biosynthesis from xylose through the phosphoketolase pathway is a carbon-efficient process (Henard et al., 2015). In the process of converting xylose from lignocellulose to ethanol, the NADH reoxidation limited the fermentation of *S. cerevisiae*. A phosphoketolase pathway was constructed by expressing genes of phosphotransacetylase and acetaldehyde dehydrogenase, and the engineered *S. cerevisiae* displayed higher ethanol yield and xylose consumption (Sonderegger et al., 2004) (Table 2).

#### 3.2.3 Microbial metabolic engineering based on pentose phosphate pathway

**Fig. 4:**
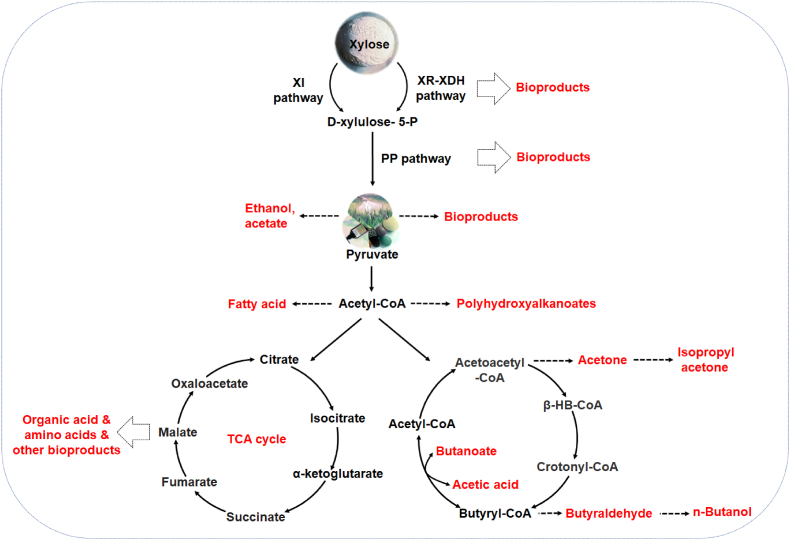
The strategies of metabolic engineering for converting xylose to value-added bioproducts. Based on the pathway and intermediates of xylose metabolism in microorganisms, different strategies have been developed for value-added bioproduct synthesis.

After xylose is converted to xylulose-5-P through XI or XR/XDH pathway, most xylulose-5-P enters the PP pathway for further metabolism. The non-oxidized PP pathway mediates xylose metabolism into the glycolytic pathway and also provides precursors for the synthesis (Fig. 4) (Chu and Lee, 2007; Hahn-Hagerdal et al., 2007; Almeida et al., 2011). It has been speculated that the limiting node factors in the PP pathway might be transketolase (Tkl1), transaldolase (Tal1), and xylulose kinase (XK) (Brat and Boles, 2013). To study the limiting metabolic steps in the utilization of xylose, different pathways were constructed in *S. cerevisiae*. The XI and XR/XDH pathway were respectively combined with the PP pathway in the engineered *S. cerevisiae*, and it was found that the expression of transaldolase (TAL), transketolase (TKL), ribose 5-phosphate ketol-isomerase (RKI), and ribulose 5-phosphate epimerase (RPE) of PP pathway promoted xylose utilization (Karhumaa et al., 2005).

The inconsistent optimal temperatures of *S. cerevisiae* and mesophilic cellulase hinders the simultaneous saccharification and fermentation (SSF) of lignocellulose for ethanol production. To increase the fermentation temperature of *S. cerevisiae* for ethanol production, the genes of PP pathway from *S. cerevisiae* and thermostable *Kluyveromyces marxianus* were co-expressed. It was found that xylose metabolism was improved by the expression of thermostable PP pathway genes, and ethanol productivity reached 0.51 g/L/h at 38 °C with engineered *S. cerevisiae* (Kobayashi et al., 2017). To increase ethanol production from xylose, the engineered *S. cerevisiae* with the expression of genes of PP pathway was further systematically optimized. It was found that *S. cerevisiae* with the expression of transketolase exhibited the highest rate of xylose consumption. The *S. cerevisiae* with the co-expression of XI and PP pathways showed higher ethanol yield from xylose than strains with XR/XDH pathway (Kobayashi et al., 2018).

The intermediates of PP pathway for xylose metabolism can also be used as a feedstock for biosynthesis, for example, sedoheptulose 7-phosphate is a key substrate for shinorine synthesis. An engineered *S. cerevisiae* was constructed by expressing genes from *Nostoc punctiform* for shinorine synthesis. Through further metabolic modulating *S. cerevisiae* and fermentation optimization, an improved shinorine production (31.0 mg/L) was obtained using xylose and glucose as substrates (Park et al., 2019). As NADPH availability is a rate-limiting factor in converting xylose to xylitol, through expressing genes of PP pathway and knocking-out Embden-Meyerhof-Parnas pathway genes in *E.coli*, the NADPH supply and xylitol production were increased. Compared with the control strain, xylitol production from corncob hydrolysates increased by 13.3% with the NADPH-enhanced strain (Yuan et al., 2021). A xylose utilization pathway was engineered in cyanobacteria *Synechocystis* sp. PCC 6803 for 3-hydroxypropionic acid synthesis under photomixotrophic conditions. In the engineered strain metabolizing xylose, the carbon flux of the oxidative PP pathway and acetyl-CoA production were improved. The engineered strain produced 14-fold higher 3-hydroxypropionic acid(3-HP) under heterologous conditions compared to the control strain under photoautotrophic conditions (Yao et al., 2022) (Table 2).

#### 3.2.4 Improving the resistance of microorganisms to inhibitors through metabolic engineering

In traditional pretreatment of biomass, the unwanted chemicals such as furfural, 5-hydroxymethylfurfural (5-HMF), organic acids, lignin derivatives, and phenols produced can inhibit the following microbial fermentation. Some natural microorganisms such as *S. cerevisiae* can tolerant the inhibitors to some extent, and the tolerance capacity can be increased through directed evolution, the genetic basis for inhibitor tolerance of *S. cerevisiae* was also analyzed (de Witt et al., 2019). Furfural and 5-HMF generated in the acidic treatment of lignocellulose can inhibit microbial metabolism. The natural *Pseudomonas putida* Fu1, *Cupriavidus basilensis*, and *Pseudomonas putida* ALS1267 can grow with furfural and 5-HMF as sole carbon sources, the metabolic pathways and genes for degrading the chemicals have been identified (Crigler et al., 2020). It has been reported that increasing cellular NADPH of microorganisms can increase the resistance to furfural and 5-HMF. Through deleting *pgi* gene of EMP pathway from ethanologenic *E. coli* SSK42, the tolerance of engineered *E. coli* to furfural and 5-HMF was increased (Jilani et al., 2020).

It has been found that spermidine can enhance tolerance of *S. cerevisiae* to inhibitors, an engineered *S. cerevisiae* was therefore constructed by expressing genes of spermidine synthesis and disrupting polyamine transport protein. The engineered *S. cerevisiae* can synthesize spermidine using lignocellulosic hydrolysates with furan derivatives and acetic acid (Kim et al., 2015b). To develop *S. cerevisiae* with high tolerance against various inhibitors, genes of trehalose-6-phosphate synthase and aldehyde reductase were expressed, and genes of neutral trehalase and trehalose degrading enzyme were knocked-out. Using the medium containing furfural and 5-HMF, the engineered *S. cerevisiae* displayed a higher ethanol yield compared with the original strain (Divate et al., 2017).

The inhibitors such as furfural and 5-HMF can also be used as platform compounds for value-added product synthesis. For example, 5-HMF was transformed to 2,5-furandicarboxylic acid (FDCA) by a syntrophic consortium of engineered *Synechococcus elongatus* and *Pseudomonas putida*. In the system, CO_2_ was fixed to sucrose by *S. elongatus*, then used for the engineered *P. putida* growth, and 5-HMF was catalyzed to FDCA (Lin et al., 2020). The transformation of 5-HMF to FDCA was also performed by engineering *Raoultella ornithinolytica*. The wild *R. ornithinolytica* BF60 can convert 5-HMF to FDCA was isolated, and the capacity was further promoted by overexpressing the aldehyde dehydrogenase gene and mutating genes of dicarboxylic acid decarboxylase and aldehyde reductase. The FDCA production of engineered *R. ornithinolytica* is 1.7 times higher than that of the wild-type strain (Hossain et al., 2017).

In the process of catalytic fast pyrolysis(CFP) of lignocellulose for biofuel production, a carbon-rich and toxic wastewater containing phenol, methanol, aromatic compounds, and furfural were produced. *Pseudomonas putida* was engineered to utilize the components, genes of dmp monooxygenase and meta-cleavage pathway were constitutively expressed, the native chaperones clpB, groES, and groEL and alcohol dehydrogenase were overexpressed, the pathways for utilization of furfural, acetone, and aromatic compounds were incorporated. The constructed *P. putida* strain can utilize 89% (w/w) of carbon in the mixture of lignocellulose CFP (Henson et al., 2021). The growth of *S. cerevisiae* is also inhibited by a high concentration of ethanol, the effect of different transporters on *S. cerevisiae* fermentation performance was analyzed by expressing the genes of different ATP-binding cassette (ABC) and major facilitator superfamily (MFS) in *S. cerevisiae*. It was found that the expression of PDR18 gene of ABC transporter can increase the ethanol tolerance of *S. cerevisiae* and ethanol production (Teixeira et al., 2012) (Table 2).

## 4 Present and prospects

As the main component of biomass, developing efficient and clean strategies to fully utilize hemicellulose is of great significance to biomass refineries. The solubilization of cellulose, hemicellulose, and lignin of biomass by thermophilic anaerobic bacteria *Caldicellulosiruptor* showed promising applications in lignocellulose degradation and the efficiency of which can be improved through metabolic engineering and bioreactor design. The partial solubilization of lignin is crucial for lignocellulose degradation, the corresponding enzymes and metabolic pathways need to be further studied (Peng et al., 2018; Kataeva et al., 2013; Straub et al., 2019; Weng et al., 2021). Transmembrane transport is a crucial step in xylose utilization, the design and engineering of efficient transmembrane transporting systems in chassis cells can further facilitate biosynthesis. Since xylose utilization is restricted by CCR effect in microorganisms, the mechanism of CCR effect in some microorganisms has not beed elucidated.

**Fig. 5:**
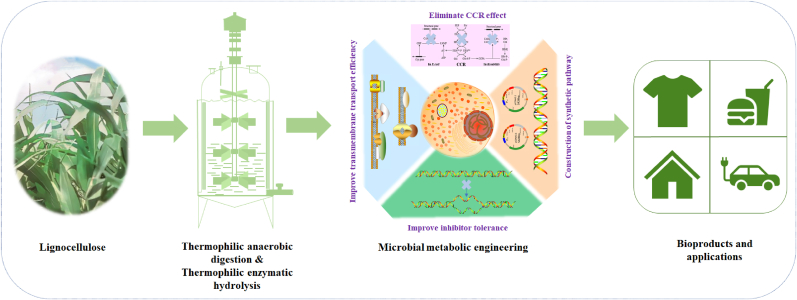
Bioprocess for converting hemicellulose of biomass into value-added bioproducts. In the bioprocesses, un-pretreated biomass is solubilized with thermophilic anaerobic bacteria or sequentially hydrolyzed with thermostable enzymes, the produced monosaccharides or organic acids were then used for following microbial fermentation and bioproducts synthesis.

In the fermentation of lignocellulosic hydrolysates for bioproduct synthesis, the inhibitors produced in the pretreatment process need to be degraded or removed. A large number of inhibitors are produced in conventional chemical or physicochemical pretreatment, the compositions of which vary depending on different pretreat conditions and compositions of lignocellulose. Although the degradation or utilization of some inhibitors has been studied, some of which such as lignin-derived chemicals still can not be fully degraded and utilized, it would be helpful to design novel pathways to metabolize the lignin-derived aromatics. A bioprocess integrating sequential biomass solubilization with thermophilic bacteria and thermostable enzymes and microbial metabolic engineering for converting xylose to value-added bioproducts were proposed (Fig. 5). Development of synthetic biology, systems biology, and metabolic engineering will help promote lignocellulose degradation, carbohydrates transmembrane transport and co-utilization, and utilize hemicellulose to produce more value-added bioproducts.

## Declaration of competing interest

The authors declare that they have no known competing financial interests or personal relationships that could have appeared to influence the work reported in this paper.

## Data Availability

No data was used for the research described in the article.
